# Polyphenol-mediated microbiome modulation in STEMI patients: a pilot study

**DOI:** 10.3389/fmed.2025.1522373

**Published:** 2025-05-21

**Authors:** Argul Issilbayeva, Shynggys Sergazy, Azamat Zhashkeyev, Alexandr Gulyayev, Samat Kozhakhmetov, Zarina Shulgau, Madiyar Nurgaziyev, Ayaulym Nurgaziyeva, Sanzhar Zhetkenev, Nurislam Mukhanbetzhanov, Zharkyn Jarmukhanov, Zhanel Mukhanbetzhanova, Elizaveta Vinogradova, Zhaxybay Zhumadilov, Almagul Kushugulova, Mohamad Aljofan

**Affiliations:** ^1^National Laboratory Astana, Center for Life Sciences, Nazarbayev University, Astana, Kazakhstan; ^2^Karaganda Medical University, Karaganda, Kazakhstan; ^3^Department of Surgery, Nazarbayev University School of Medicine, Astana, Kazakhstan; ^4^Department of Biomedical Sciences, Nazarbayev University School of Medicine, Astana, Kazakhstan

**Keywords:** gut microbiome, STEMI, polyphenol, 16S rRNA sequencing, TMAO

## Abstract

**Introduction:**

This study investigates the effects of polyphenol supplementation on gut microbiome composition and cardiovascular health in patients with ST-segment elevation myocardial infarction (STEMI).

**Methods:**

Double-blind randomized control trial where participants received either polyphenol supplementation or placebo for 3 months, after which composition of the gut microbiome; clinical and laboratory parameters, including TMAO levels and oxidative stress levels, were assessed.

**Results:**

The stable TMAO levels (from 0.5 [0.2–0.9] to 0.4 [0.3–0.9] μmol, *p* > 0.05) were observed in the polyphenol group, compared to the increase observed in the placebo group (from 0.5 [0.3–0.6] to 0.7 [0.5–1.4] μmol, *p* < 0.001). Polyphenol supplementation significantly decreased the *Firmicutes*/*Bacteroidetes* ratio (*p* = 0.04) and increased beneficial bacteria such as Roseburia (*p* = 0.01), *Agathobaculum* sp. (*p* = 0.004), *Alistipes finegoldii* (*p* = 0.04) and *Sellimonas* (*p* = 0.002). Predicted metabolic pathways analysis supports potential mechanisms linking polyphenol intake to microbiome modulation and TMAO regulation.

**Conclusion:**

Our findings demonstrate that polyphenol supplementation maintains stable TMAO levels by restructuring gut microbiome composition in STEMI patients, evidenced by a more focused microbiome with a significant increase in beneficial butyrate-producing bacteria (*Roseburia, Agathobaculum* sp., *Alistipes finegoldii*, and *Sellimonas*) and a decreased *Firmicutes*/*Bacteroidetes* ratio, suggesting microbiome-mediated cardioprotective effects. While promising,l our preliminary findings require further studies with larger cohorts and more advanced sequencing methods to establish their significance for cardiovascular health.

**Clinical trial registration:**

ClinicalTrial.gov, identfier: NCT06573892.

## Introduction

The gut-heart axis is a new concept that offers fresh perspectives in cardiology practice (1). The gut microbiome represents the most diverse and elaborate collection of microorganisms in the human body, and it plays a vital role in maintaining health and influencing disease (1–3). The specific ways the microbiome is linked to cardiovascular disease (CVD) development and progression are not fully understood. Metabolites linked to gut microbiota contribute to the development of CVD (4), including ST-segment elevation myocardial infarction (STEMI) (5–8), one of the most frequent cardiovascular disorders with a marginally surging morbidity and mortality rate on a global scale (9). Specifically, TMAO is a liver-derived metabolite formed from trimethylamine (TMA), which is produced by gut microbes from dietary precursors such as L-carnitine, substrate for microbial metabolism. TMA is absorbed into the bloodstream and oxidized by hepatic flavin-containing monooxygenases (primarily FMO3) to form TMAO (2). According to existent data, TMAO can be used to predict major adverse cardiac events (MACE) in the short and long term (10, 11) and is suggested as a prognostic biomarker in STEMI patients (8, 12). TMAO disrupts reverse cholesterol transport and cholesterol breakdown, which leads to an increase in foam cell formation (13). Furthermore, it also impairs cholesterol clearance by suppressing bile acid synthesis (14). Notably, gut bacteria capable of producing TMAO are mainly from the *Firmicutes* phyla (15, 16) and the *Firmicutes/Bacteroidetes* ratio is being proposed as a predictor of TMAO concentration (17).

Plant-derived polyphenols interact bidirectionally with a gut microbiome, influencing their composition while being transformed into bioactive metabolites (13, 18). The gut-heart axis concept suggests gut dysbiosis contributes to cardiovascular pathogenesis, with polyphenols potentially restoring microbial balance (13). These compounds exhibit antioxidant, anti-inflammatory, and anti-diabetic properties, potentially reducing cardiovascular event risk (19–25), including myocardial infarction (26). Accumulating evidence indicates that polyphenols can positively modulate the gut microbiome in atherosclerosis by enhancing bacterial diversity, promoting the growth of beneficial genera such as *Akkermansia, Parabacteroides, Ruminococcus, Anaerostipes, Anaerotruncus, Bacteroides, Lactobacillus*, and *Bifidobacterium*, which collectively contribute to improved intestinal barrier function and reduced systemic inflammation (27). Polyphenols demonstrate promising potential in managing cardiovascular disease risk through TMAO reduction by modulating gut microbiota and metabolic pathways. These compounds can modify bacterial populations, inhibiting bacterial strains responsible for TMAO precursor metabolism, potentially improving lipid profiles, blood pressure and endothelial function, enhancing nitric oxide production, reducing platelet aggregation, slowing atherosclerosis progression and reducing stenosis severity (2, 28–31). However, evidence from existing clinical trials studying polyphenol's cardiovascular effects varies across different studies. There is also a discrepancy between low-dose alcohol-containing wine studies and high-dose supplement trials, which creates inconsistency (32), warranting further investigations.

Molecular pathways and biochemical processes through which polyphenols modulate gut microbial populations in CVD need to be understood. Thus, the current study aims to examine polyphenol-induced changes in the gut microbiome and their relationship with STEMI. This will improve our understanding of polyphenols' potential to improve cardiovascular health.

## Methods

### Ethical approval

The present study was approved by the Local Ethical Commission of the National Laboratory Astana- Nazarbayev University on 24/09/2020, Approval No. 05-2020. The study aims, objectives, risks, and protocols were meticulously explained to all participants, and they were provided with leaflets containing relevant information before signing the informed consent form.

### Recruitment

Patients were recruited through Karaganda Cardiac Hospital, “Multidisciplinary Hospital No. 2” in Kazakhstan, based on their medical diagnosis and patient profile, during the period from May 2022 to September 2023. The study was registered on ClinicalTrial.gov, ID: NCT06573892.

### Inclusion criteria

The inclusion criteria were patients (males and females) over 18 years of age, diagnosed with STEMI, who underwent primary percutaneous coronary intervention within 12 h after the onset of the disease. The diagnosis of STEMI was established based on clinical symptoms—chest pain lasting more than 20 min in combination with electrocardiographic changes (ST-segment elevation ≥ 1 mV in at least two adjacent leads (or a blockage of the left leg of the Gis bundle for the first time) and an increase in Creatine kinase and troponin levels.

### Exclusion criteria

The exclusion criteria were patients with cardiogenic shock, ineffective results of stenting of infarct-dependent arteries (weak filling or lack of filling of arteries), chronic intestinal diseases, or acute intestinal diseases at the screening time. Also excluded patients were those with dietary restrictions such as allergies or intolerance to grapes and those with clinically significant infections in the 6 months prior to the start of the study. For example, patients with infections requiring hospitalization or parenteral antimicrobial therapy, opportunistic infections or the presence of anamnesis of any disease for which (according to the researcher's assessment) an exacerbation is possible due to participation in the study. Other exclusion criteria include a history of oncological or lymphoproliferative disease 5 years before the baseline visit, psychiatric disorders, including recent (within the last year) or active suicidal thoughts, or patients who require additional coronary artery revascularization [Coronary Artery Bypass Grafting(CABG) or stenting].

### Randomization

Patients were randomized according to the type of dietary intervention by an independent research assistant who was not involved in any other aspect of the study. Participants were randomly assigned to one of the experimental groups (1:1) in sets of 6, utilizing the resource at https://www.sealedenvelope.com/simple-randomiser/v1/lists. The packets, which included either a polyphenol or a placebo, were pre-packaged based on the randomization scheme. As a result, participants, medical staff, and evaluators did not know which group each participant was in until the data was examined and the database was accessed.

### Polyphenol supplement

Dietary intervention was carried out on the background of standard therapy after the acute phase of STEMI. The first group was prescribed daily for 3 months, no later than 30 minutes before meals, 15 ml of a concentrate of grape-extracted polyphenols, and the second group was prescribed a placebo. The polyphenol supplement used in this study has an established favorable safety profile. It conforms to all applicable state standards and regulations (Eurasian Economic Union Committee for Sanitary and Epidemiological Control Certificate No KZ.16.01.98.003.R.001043.11.21). Safety testing was conducted in an accredited laboratory for food product analysis (Nutritest No KZ.T.02.E0177). The only potential side effect of concern is a reaction to grape components, which was considered in the exclusion criteria. The placebo consisted of water with grape aromatizer and caramel coloring (E150) added at a low concentration to match the appearance of the polyphenol concentrate.

### Examination

Participants were assessed at baseline (M0) and after 3 months of polyphenol intake (M3). Blood plasma and stool samples were collected from all study participants during hospitalization after primary percutaneous coronary intervention upon discharge from the hospital and after 3 months of intervention. In addition, clinical parameters were analyzed, including complete blood count (CBC); plasma lipid profile (including low-density lipoproteins (LDL); cholesterol (TC) and triglyceride (TG) levels; cardiac troponin I (cTnI); kidney function, including plasma creatinine; blood urea nitrogen (BUN); glucose (GLU); total protein (TPro); alanine transaminase (ALT); aspartate transferase AST; total bilirubin (TBil); creatine kinase-myocardial band (CK); urine test; electrocardiography and echocardiography.

Synergy between percutaneous coronary intervention with TAXus and cardiac surgery (SYNTAX) evaluation was conducted on all participants at the beginning of the study. Two interventional cardiologists (unaware of the TMAO results and clinical outcomes) evaluated the SYNTAX score using an online calculator (https://www.syntaxscore.com, version 2.28). The SYNTAX index was defined as the SYNTAX of the acute phase—the residual SYNTAX of the chronic phase, and the plaque progression group was defined as the highest tertile on the SYNTAX scale (≥3). For long-term risk prediction after percutaneous coronary intervention (PCI), the Logistic Clinical SYNTAX Score (LCSS) was assessed at the beginning of the study (33). All participants underwent an evaluation of the risk of developing CVD using Systematic Coronary Risk Evaluation 2 (SCORE2) at the M0 and M3. The risk was assessed as low, moderate and high (34). All participants received dietary guidance in accordance with Mediterranean diet and Dietary Approaches to Stop Hypertension (DASH) nutritional recommendations in accordance with the Republic of Kazakhstan's clinical protocol for STEMI patient management (35).

### TMAO measurement

Quantitative determination of TMAO concentration in plasma samples was conducted utilizing a combination of high-performance liquid chromatography and tandem mass spectrometry (HPLC-MS/MS). The analytical instrumentation consisted of an Agilent 1260 Infinity chromatographic system coupled with a G6130A quadrupole mass spectrometer (both manufactured by Agilent Technologies, Santa Clara, CA, USA).

Blood specimens were collected in ethylenediaminetetraacetic acid (EDTA)—containing tubes and underwent immediate centrifugation (3,000 rpm, 15 min) to isolate plasma, which was subsequently cryopreserved at −80°C until analysis. The analytical protocol employed several high-purity reagents: 95% trimethylamine N-oxide standard, 95% formic acid, and 99.9% acetonitrile (all sourced from Sigma-Aldrich, St. Louis, MO, USA), along with ultrapure water (18.2 mg/L resistivity) generated using the Milli-Q purification system (Millipore, Burlington, MA, USA).

The sample preparation protocol involved protein precipitation by combining 100 μL of thawed plasma with 600 μL acetonitrile, followed by high-speed centrifugation (20,000 × g, 10 min) at 4°C. The resulting supernatant (100 μL) was diluted with an equal volume of water, and 10 μL of this preparation was injected into the HPLC-MS/MS system.

Chromatographic separation was achieved using a ZORBAX Eclipse XDB C-18 analytical column (2.1 × 75 mm, 3.5 μm particle diameter) with a corresponding guard column (12.5 × 4.6 mm, 3.5 μm) maintained at 80°C. The separation employed an isocratic elution method using a binary mobile phase system: solution A (0.125% formic acid in a 1:1 acetonitrile-water mixture) and solution B (0.125% formic acid in a 1:1 water mixture). The chromatographic conditions included a constant flow rate of 0.250 mL/min at a column temperature of 30°C. TMAO concentrations were determined by integrating the chromatographic peak areas from the resulting analytical curves.

### Oxidative stress measurement

The oxidant status (the potential of the body's antioxidant systems) was determined in blood plasma samples by a rapid test using the e-BQC apparatus (BioQuoChem-BQC redox technologies, Oviedo, Spain) with an assessment of the total antioxidant potential and the antioxidant capacity of fast and slow antioxidants (24). We immediately received results of fast (Q1) and slow (Q2) active antioxidants, as well as total charge (QT). According to standard curves, this data is further translated into CEAC (equivalent to vitamin C antioxidant capacity).

### Microbiome examination

The composition of the gut microbiome was determined from stool samples. The collection of biomaterial (stool) for the gut microbiome analysis was carried out in DNA/RNA Shield™ test tubes, catalog number R1101 (Zymo Research). DNA extraction was performed using the ZymoBIOMICS™ DNA extraction kit according to the recommended protocol.

### Sequencing data

The 16S locus libraries were prepared using NEXTflex^®^ 16S V1-V3 Amplicon-SeqKit (Perkin Elmer, catalog number NOVA-4202-04). Amplicons were sequenced on the MiSeq device (Illumina). Analysis: Demultiplexing, filtering, and determination of amplicon sequence variant (ASV) and taxonomic identification were performed using the LotuS program. After the abundance filtering at M0, the polyphenol group remained 36, in the placebo group 41 samples, respectively; at M3, the polyphenol group remained 25, and in the placebo group 25 samples. The 16S amplicon sequencing data, starting from raw reads, underwent taxon density tables processing using the Less OTU Scripts 2 (LotuS2) pipeline. Critical steps in this process included demultiplexing, quality filtering, and dereplication of reads, which were carried out with the assistance of a straightforward demultiplexer (sdm). Additionally, chimeric sequences were identified and eliminated using UCHI Marker Examiner (UCHIME) algorithms. Taxonomic postprocessing and sequence clustering, employing the combined databases such as SILVA, Greengenes 2 (GG2), and Human Intestinal Tract database (HITdb), were performed using Lowest Common Ancestor (LCA) and Divisive Amplicon Denoising Algorithm 2 (DADA2) sequence clustering algorithms, respectively (36). The total number of reads accounted for 28,147,916, employing a similarity threshold of 97% for distance comparison. After filtering, the sequences were categorized into 7754 ASVs and were attributed to the bacterial domain, with 25,637,043 reads remaining in the matrix.

### Metabolic pathways

To further investigate the functional potential of the microbial community, metabolic pathway analysis was performed using Phylogenetic Investigation of Communities by Reconstruction of Unobserved States (PICRUSt2) version 2.5.0. Following default settings, PICRUSt2 predicted functional metagenomic profiles based on the 16S rRNA sequencing data. To determine the gene family copy numbers for each amplicon sequence variant (ASV), a reference tree with a Nearest Sequenced Taxon Index (NSTI) threshold of 2 was used.

### Statistical methods

Statistical analysis of results was performed on R v4.4.1 and Python v3.9.14. Independent *T*-test and Mann–Whitney *U-*test were used to compare groups. Tests were automatically selected based on normality and homoscedasticity criteria. The Shapiro and Levene tests were used to test these criteria. α and β diversity were evaluated using vegan v2.6.1 and OTUtable v1.1.2. Within-sample diversity was assessed using Shannon and Chao1 indices. Between-sample diversity was evaluated using Bray-Curtis metrics on Hellinger-transformed data. Differential analysis on taxonomic data was performed using Linear discriminant analysis Effect Size (LEfSe) v1.10.0 (LDA > 2 and *p* ≤ 0.05) from the microbiome Marker package and STAMP v2.1.3 on functional data. Only features with at least 30% prevalence were retained for the analysis. The correlation was computed using Pearson's r coefficient from SciPy v1.10.1. Right-hand outliers in the clinical data were replaced by 95%. Compositional data was transformed using the centered log-ratio (clr) method, and all other variables were standard scaled. All multiple comparisons were adjusted using the False Discovery Rate with Benjamini–Hochberg (FDR BH) method, and the significance level was set at 0.05. No numerous comparison adjustments were applied when comparing significantly differentially abundant parameters. Visualization was performed with ggplot2 v3.5.1, Matplotlib v3.7.1 and seaborn v0.11.2. The two-sided value of *p* < 0.05 was considered statistically significant.

## Results

### Randomization

**Figure 1:**
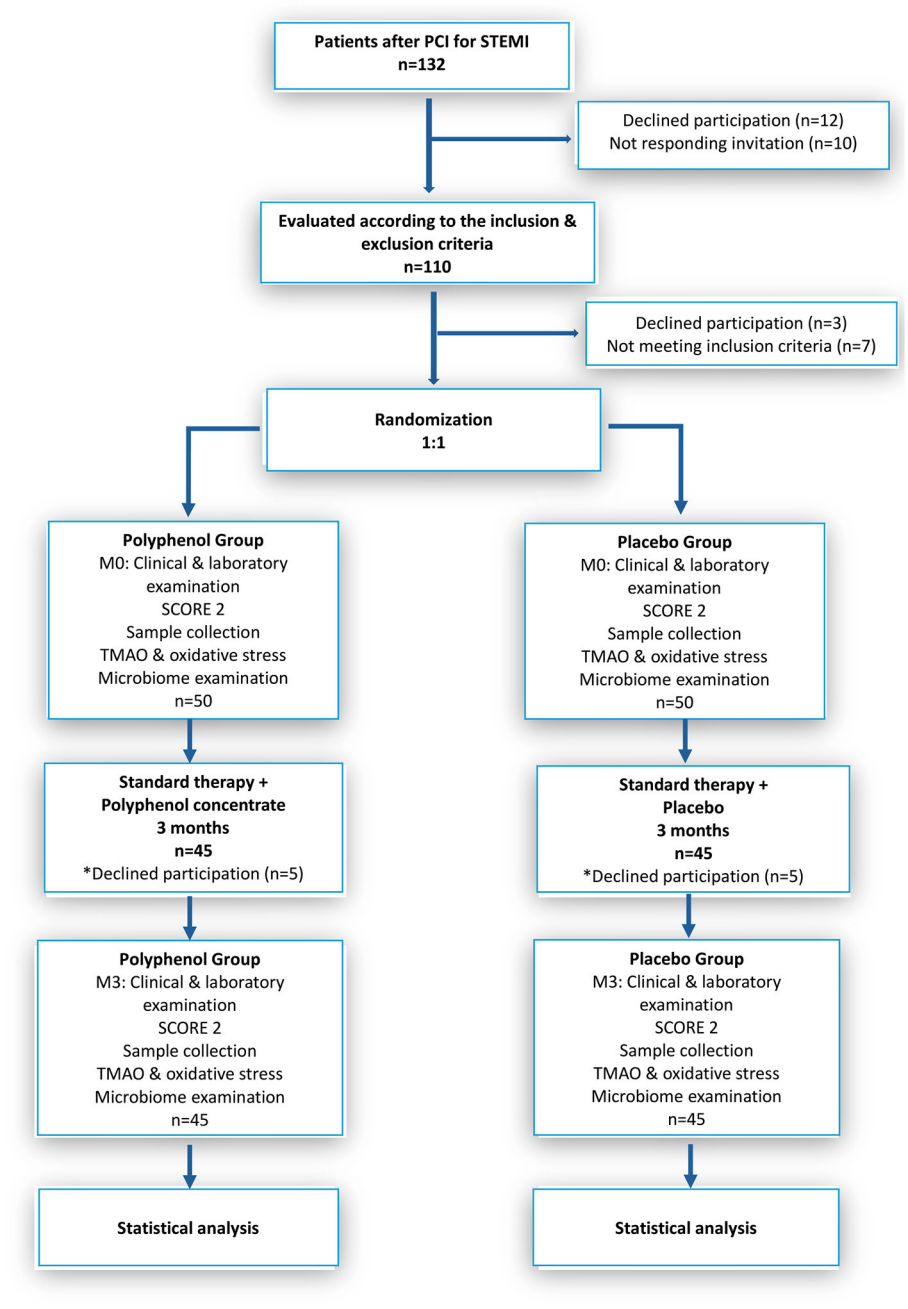
The scheme of the study designed by CONSORT. PCI, percutaneous coronary intervention; STEMI, ST-elevation myocardial infarction; TMAO, Trimethylamine N-oxide; M0-baseline; M3-after 3 months polyphenol intake. *Additional information.

In total, 132 patients were identified and invited to participate in the study; 12 declined to participate, and 10 did not respond. The remaining 110 patients underwent initial screening, after which three refused to continue in the study, and seven failed the inclusion criteria. The included patients were randomized into two groups of 50 each; the first group received polyphenol concentrate and the second placebo, respectively. However, five more patients in each group were lost as they either declined to continue or did not comply (Figure 1). A total of 45 participants from both study groups completed all study visits through month 3 (M3).

### Demographic data

There was no significant difference in the average age of each group, with 64.0 [55.0–66.0] and 61.0 [55.0–68.0], *p* = 0.83, respectively. Similarly, there were no significant differences in gender, ethnicity, social status, and other disease-related characteristics such as smoking, BMI, SYNTAX Score I, LCSS, Score 2, cTnL, and Main diagnosis and accompanied comorbidities, *p* > 0.05 (Table 1).

**Table 1:** Clinical-demographic data of study groups at the baseline.

**Parameters**	**Placebo (*n =* 45)**	**Polyphenol (*n =* 45)**	***p***
Gender, *n*	32/13	25/20	0.19^a^
BMI, M [IQR]	30.1 [26.1–32.7]	28.4 [25.1–31.0]	0.10^b^
Weight, kg, M [IQR]	87.0 [74.0–95.0]	77.0 [70.0–90.0]	0.06^b^
Height, cm, M [IQR]	168.0 [165.0–176.0]	165.0 [160.0–174.0]	0.36^b^
cTnI, ng/ml, M [IQR]	1.1 [0.11–3.1]	0.3 [0.07–1.47]	0.11^c^
CRCL, ml/min, M [IQR]	91.0 [83.0–103.0]	90.0 [82.0–97.0]	0.41^c^
SYNTAX_Score_I, M [IQR]	11.0 [8.0–20.5]	11.0 [8.0–17.0]	0.56^c^
LCSS, %, M [IQR]	1.8 [1.1–3.2]	1.7 [1.1–3.0]	0.97^c^
Non Smoking/Smoking, *n*	31/14	29/15	0.94^d^
Race Mongoloid/Europioid, *n*	15/30	16/29	1.0^d^
Social_status: Disabled/Disabled gr2/Disabled gr3/Employed/Retired/Unemployed, *n*	0/1/1/20/20/3	1/0/2/22/15/5	0.60^d^
Main.Diagnosis, STEMI LAD/RCA/CX, *n*	18/23/3	19/20/5	0.69^d^
Comorbidities/No comorbidities, *n*	27/18	24/21	0.67^d^
DM, *n*	8/37	5/40	0.55^a^
Arrhythmia, *n*	3/42	3/42	1.0^a^
AH_III, *n*	14/31	15/30	1.0^a^
COPD	3/42	1/44	0.62^a^
Anemia	2/43	2/43	1.0^a^
SCORE 2, %, M [IQR]	24.2 ± 11.2	24.3 ± 11.4	0.96^b^

a Fisher's exact test,

b Ind. T-test,

c Mann–Whitney U-test,

d Chi^2^.

BMI, Body Mass Index; cTnI, Cardiac Troponin I; CRCL, Creatinine Clearance; SYNTAX_Score_I, Synergy between Percutaneous Coronary Intervention with Taxus and Cardiac Surgery Score; LCSS, Left ventricular Circumferential Systolic Strain; STEMI, ST-Elevation Myocardial Infarction; LAD, Left Anterior Descending coronary artery; RCA, Right Coronary Artery; CX, Circumflex coronary artery; DM, Diabetes Mellitus; AH_III, n-Arterial Hypertension stage III; COPD, Chronic Obstructive Pulmonary Disease; SCORE 2, Systematic Coronary Risk Evaluation 2.

### Biochemical analyses

Intergroup comparative analysis of clinical and laboratory characteristics at baseline (M0) and 3-month (M3) follow-up demonstrated statistically significant differences in TMAO levels that were reduced by half after 3 months in the polyphenol group, *p* < 0.01 compared to placebo (Table 2). The LDL level was high in the polyphenol group compared to placebo at the start of the experiment, *p* = 0.005 and remained unchanged (Table 2 and Supplementary Table S1).

**Table 2:** Intergroup analysis of clinical and laboratory parameters: polyphenol vs. placebo comparison at baseline (M0) and 3-month (M3) follow-up.

**Parameters**	**Timepoint**	**Placebo**	**Polyphenol**	***p***
TMAO, mol, M [IQR]	M0	0.5 [0.3–0.6]	0.5 [0.2–0.9]	0.7^b^
	M3	0.7 [0.5–1.4]	0.4 [0.3–0.9]	<0.01^b^
AST, U, M [IQR]	M0	31.0 [23.0–56.0]	32.0 [25.0–44.0]	0.93^b^
	M3	27.0 [21.0–42.0]	25.0 [20.0–32.0]	0.15^b^
ALT, U, M [IQR]	M0	35.0 [20.0–54.0]	36.0 [23.0–62.0]	0.35^b^
	M3	28.0 [20.0–44.0]	27.0 [21.0–38.0]	0.49^b^
GLU, mol, M [IQR]	M0	6.5 [5.3–7.7]	6.0 [5.5–8.3]	0.89^b^
	M3	5.7 [4.9–7.0]	5.9 [5.1–7.1]	0.41^b^
TC, mol, M [IQR]	M0	4.5 [3.6–5.23]	4.5 [3.9–5.4]	0.94^a^
	M3	4.3 [3.2–4.9]	4.4 [3.9–5.1]	0.22^a^
TG, mol, M [IQR]	M0	1.0 [1.0–1.5]	1.1 [1.1–1.3]	0.15^b^
	M3	1.1 [0.8–1.5]	1.3 [1.1–1.3]	0.20^b^
LDL, mol, M [IQR]	M0	0.36 [0.36–0.4]	0.41 [0.38–0.54]	<0.01^b^
	M3	0.4 [0.39–0.5]	0.41 [0.38–0.6]	0.34^b^
CK, U, M [IQR]	M0	112.0 [89.0–248.0]	115.0 [84.0–156.0]	0.22^b^
	M3	106.0 [80.0–187.0]	92.0 [73.0–128.0]	0.12^b^
Q1.C, mC, M [IQR]	M0	11.7 [10.1–14.2]	11.6 [10.0–13.3]	0.64^b^
	M3	10.9 [9.0–14.2]	11.4 [10.2–13.4]	0.25^b^
Q2.C, mC, M [IQR]	M0	8.2 [7.3–9.2]	8.0 [7.5–10.2]	0.68^b^
	M3	7.9 [6.9–8.8]	8.0 [7.5–9.4]	0.11^b^
QT.C, mC, M [IQR]	M0	19.0 [16.8–23.4]	19.4 [17.4–22.2]	0.90^a^
	M3	18.6 [16.0–23.3]	20.1 [17.9–23.0]	0.09^b^
Echo. F, %, M [IQR]	M0	48.0 [44.0–53.0]	46.0 [44.0–52.0]	0.86^b^
	M3	50.0 [46.0–56.0]	50.0 [45.0–56.0]	0.61^a^
SCORE 2, %, M [IQR]	M0	22.0 [16.0–33.0]	22.0 [17.0–33.0]	0.96^a^
	M3	14.0 [9.0–21.0]	12.0 [9.0–19.0]	0.37^b^

a Ind. T-test,

b Mann–Whitney U-test.

TMAO, Trimethylamine N-oxide; AST, Aspartate Aminotransferase; ALT, Alanine Aminotransferase; GLU, Glucose; TC, Total Cholesterol; TG, Triglycerides; LDL, Low-Density Lipoprotein; CK, Creatine Kinase; Q1, C-Q wave duration corrected; Q2, C-Q wave amplitude corrected; QT.C, QT interval corrected; Echo. F, Ejection Fraction; SCORE 2, Systematic Coronary Risk Evaluation 2.

Analysis of clinical and laboratory parameters from baseline (M0) to 3 months (M3) revealed a significant two-fold increase in TMAO levels in the placebo group (from 0.5 [0.3–0.6] to 0.7 [0.5–1.4], *p* < 0.001; Table 3), while TMAO levels remained stable in the polyphenol group (from 0.5 [0.2–0.9] to 0.4 [0.3–0.9], *p* > 0.05). Interestingly, the AST and ALT levels decreased in the polyphenol group from M0 to M3, *p* = 0.007 and *p* = 0.03, respectively. The LDL level was elevated in the placebo group from M0 to M3, *p* = 0.004. The ejection fraction slightly increased in both study groups, *p* ≤ 0.05 (Table 3).

**Table 3:** Intragroup analysis of clinical and laboratory parameters: baseline (M0) vs. 3-month (M3) comparison in polyphenol and placebo groups.

**Parameters**	**Group**	**M0**	**M3**	***p***
TMAO, mol, M [IQR]	Placebo	0.5 [0.3–0.6]	0.7 [0.5–1.4]	<0.001^b^
	Polyphenol	0.5 [0.2–0.9]	0.4 [0.3–0.9]	0.97^b^
AST, U, M [IQR]	Placebo	31.0 [23.0–56.0]	27.0 [21.0–42.0]	0.27^b^
	Polyphenol	32.0 [25.0–44.0]	25.0 [20.0–32.0]	<0.01^b^
ALT, U, M [IQR]	Placebo	35.0 [20.0–54.0]	28.0 [20.0–44.0]	0.70^b^
	Polyphenol	36.0 [23.0–62.0]	27.0 [21.0–38.0]	<0.05^b^
GLU, mol, M [IQR]	Placebo	6.5 [5.3–7.7]	5.7 [4.9–7.0]	0.08^b^
	Polyphenol	6.0 [5.5–8.3]	5.9 [5.1–7.1]	0.24^b^
TC, mol, M [IQR]	Placebo	4.5 [3.6–5.2]	4.3 [3.2–4.9]	0.15^a^
	Polyphenol	4.5 [3.9–5.4]	4.4 [3.9–5.1]	0.59^a^
TG, mol, M [IQR]	Placebo	1.0 [1.0–1.5]	1.1 [0.8–1.5]	0.36c
	Polyphenol	1.1 [1.1–1.3]	1.3 [1.1–1.3]	0.17^b^
LDL, mol, M [IQR]	Placebo	0.4 [0.3–0.4]	0.4 [0.3–0.5]	<0.01^b^
	Polyphenol	0.4 [0.3–0.5]	0.4 [0.3–0.6]	0.64^b^
CK, U, M [IQR]	Placebo	112.0 [89.0–248.0]	106.0 [80.0–187.0]	0.31^b^
	Polyphenol	115.0 [84.0–156.0]	92.0 [73.0–128.0]	0.10^b^
Q1, C, M [IQR]	Placebo	11.7 [10.1–14.2]	10.9 [9.0–14.2]	0.13^a^
	Polyphenol	11.6 [10.0–13.3]	11.4 [10.2–13.4]	0.95^b^
Q2, C, M [IQR]	Placebo	8.2 [7.3–9.2]	7.9 [6.9–8.8]	0.24^b^
	Polyphenol	8.0 [7.5–10.2]	8.0 [7.5–9.4]	0.91^b^
QT, C, M [IQR]	Placebo	19.0 [16.8–23.4]	18.6 [16.0–23.3]	0.21^b^
	Polyphenol	19.4 [17.4–22.2]	20.1 [17.9–23.0]	0.45^b^
Echo. F, %, M [IQR]	Placebo	48.0 [44.0–53.0]	50.0 [46.0–56.0]	<0.05^a^
	Polyphenol	46.0 [44.0–52.0]	50.0 [45.0–56.0]	<0.05^a^
SCORE 2, %, M [IQR]	Placebo	22.0 [16.0–33.0]	14.0 [9.0–21.0]	0.51^b^
	Polyphenol	22.0 [17.0–33.0]	12.0 [9.0–19.0]	0.66^b^

a Ind. T-test,

b Mann–Whitney U-test.

TMAO, Trimethylamine N-oxide; AST, Aspartate Aminotransferase; ALT, Alanine Aminotransferase; GLU, Glucose; TC, Total Cholesterol; TG, Triglycerides; LDL, Low-Density Lipoprotein; CK, Creatine Kinase; Q1, C-Q wave duration corrected; Q2, C-Q wave amplitude corrected; QT, C-QT interval corrected; Echo. F, Ejection Fraction; SCORE 2, Systematic Coronary Risk Evaluation 2.

There were significant differences in blood and urine tests; monocyte levels in blood and leucocytes in urine decreased in both study groups over the period, *p* ≤ 0.05. The urine pH decreased in the polyphenol group at M3 compared to M0, *p* = 0.007. Other parameters did not demonstrate any differences at the baseline and after 3 months (Supplementary Table S3).

### Microbiome analysis

**Figure 2:**
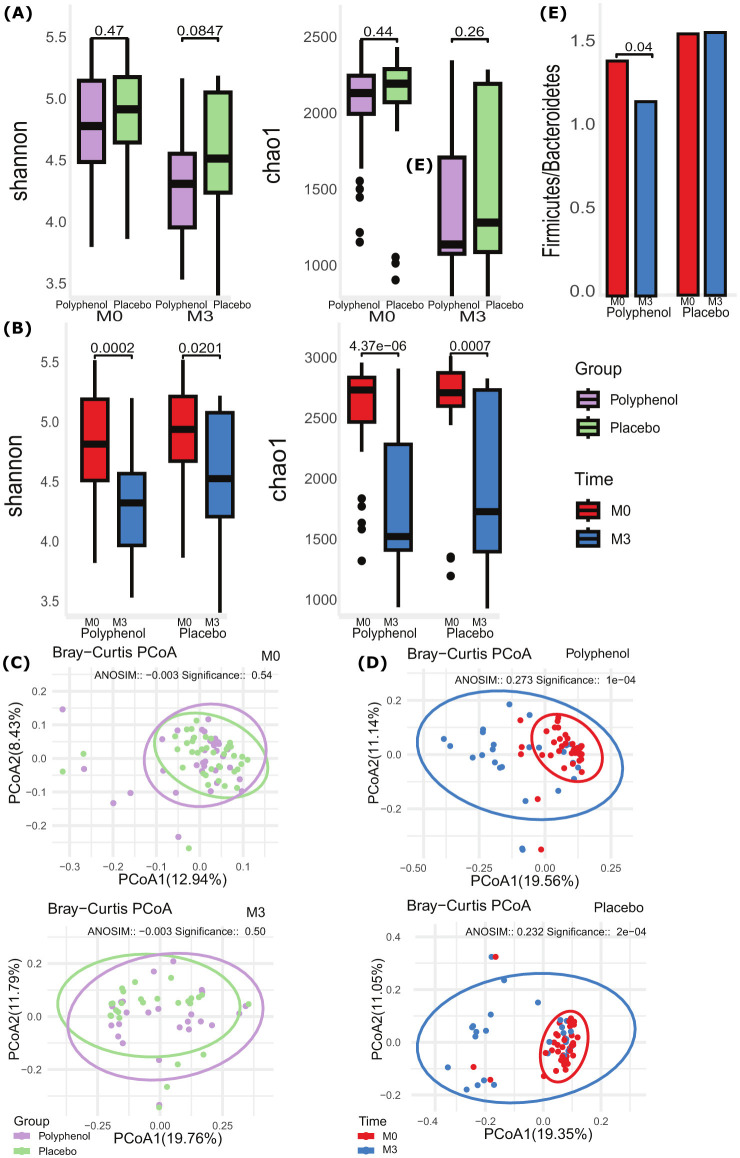
Changes in gut microbiome diversity and composition following polyphenol supplementation in STEMI patients. **(A)** α diversity measures: Shannon index (left) and Chao1 index (right) at baseline (M0) and after 3 months (M3) comparing Polyphenol and Placebo groups. **(B)** α diversity measures: Shannon index (left) and Chao1 index (right) at baseline (M0) and after 3 months (M3) for Placebo (left) and Polyphenol (right) groups separately. **(C)** β diversity analysis: Bray-Curtis PCoA plot comparing Polyphenol and Placebo groups at baseline (M0) and after 3 months (M3). **(D)** β diversity analysis: Bray-Curtis PCoA plots showing changes in microbial communities from baseline (M0) to 3 months (M3) for Polyphenol and Placebo groups separately. **(E)** *Firmicutes*/*Bacteroidetes* ratio comparison between Polyphenol and Placebo groups at baseline (M0) and after 3 months (M3). The significance of grouping for beta diversity measures is tested using ANOSIM with 999 permutations. The significance level for all tests is set at *p* ≤ 0.05.

Analysis of α diversity showed no significant differences between the study groups at baseline (M0) and 3-month (M3) follow-up (*p* > 0.05) (Figure 2A). Yet, significant differences were found in both Shannon and Chao1 indexes during intragroup analysis (baseline M0 vs. 3-month M3 comparison) in both Polyphenol and Placebo groups, with α diversity decreased at 3 months compared to baseline (Shannon index: *p* = 0.0002 and *p* = 0.021; Chao1 index: *p* ≤ 0.0001 and *p* = 0.0007, respectively), (Figure 2B).

Similarly, analysis of β diversity by the Bray-Curtis index did not reveal any compositional differences between the study groups at baseline (M0) and 3-month (M3) follow-up (*p* > 0.05, (Figure 2C). Meanwhile, significant intragroup differences (baseline M0 vs. 3-month M3 comparison) were observed, with distinct clusters formed by PCoA using Bray-Curtis dissimilarity (ANOSIM: *R* = 0.27, *p* ≤ 0.0001 for the Polyphenol group; *R* = 0.23, *p* ≤ 0.001 for Placebo group). The first two principal coordinates explained 30.7% of the total variance (PCoA1: 19.56%, PCoA2: 11.14%) in the Polyphenol group and 30.4% (PCoA1: 19.35%, PCoA2: 11.05%) in the Placebo group (Figure 2D). The *Firmicutes*/*Bacteroidetes* (*F*/*B*) ratio did not experience statistically significant shifts in the placebo group between M0 and M3, *p* = 0.826, and between two study groups at M0, *p* = 0.6540, and M3, *p* = 0.141. However, there was a significant decrease in the *F*/*B* ratio in the polyphenol group between M0 and M3, *p* = 0.04 (Figure 2E).

**Figure 3:**
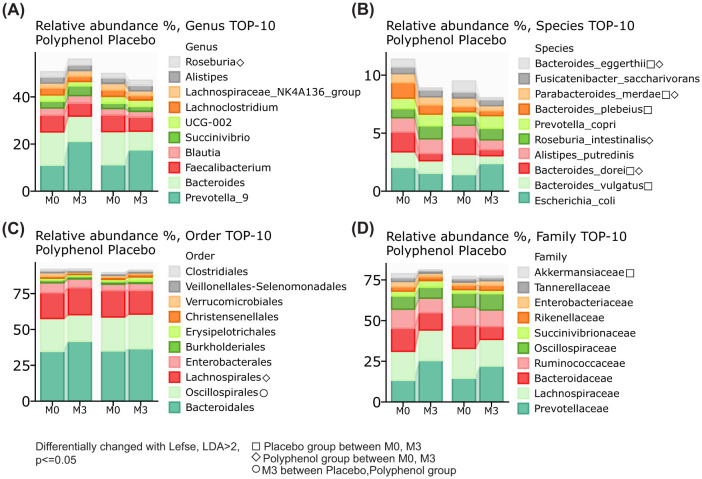
Gut microbiome composition changes following polyphenol supplementation in STEMI patients. **(A)** Relative abundance (%) of the top 10 bacterial genera at baseline (M0) and after 3 months (M3) in both Polyphenol and Placebo groups. Stacked bar plots show the distribution of genera. **(B)** Relative abundance of the top 10 bacterial species at M0 and M3 in both groups. Stacked bar plots display the distribution of species. **(C)** Relative abundance (%) of the top 10 bacterial orders at M0 and M3 in both groups. Stacked bar plots show the distribution of orders. **(D)** Relative abundance (%) of the top 10 bacterial families at M0 and M3 in both groups. Stacked bar plots display the distribution of families. The figure also includes a note about differential changes identified using LEfSe analysis (Linear discriminant analysis Effect Size) with LDA > 2 and *p* ≤ 0.05. These changes are categorized into three groups: Changes within the Placebo group between M0 and M3, Changes within the Polyphenol group between M0 and M3, and Differences between Placebo and Polyphenol groups at M3.

Most statistically significant changes in relative abundance were observed at the species level: *Bacteroides dorei* (polyphenol group *p* = 0.001, placebo group *p* = 0.007), *Bacteroides eggerthii* (polyphenol group *p* = 0.03, placebo group *p* = 0.002), and *Parabacteroides merdae* (polyphenol group *p* = 0.04, placebo group *p* = 0.02), decreased significantly in both study groups, between M0 and M3, while the abundance *Bacteroides plebeius* (*p* = 0.03) and *Bacteroides vulgatus* (*p* = 0.001) noticeably decreased in placebo group between M0 and M3. *Roseburia* significantly increased in the polyphenol group between M0 and M3 at the genus level, *p* = 0.01 (Figure 3A). At the family level, Akkermansiaceae significantly decreased in the Placebo group, between M0 and M3, *p* = 0.03, and at the order level, Lachnospirales were elevated in the Polyphenol group between M0 and M3, *p* = 0.01, and Oscillospirales was significantly higher in Placebo group compared to Polyphenol group at M3, *p* = 0.002 (Figures 3B–D). Refer to Supplementary Tables S3–S5 for *p*-values corresponding to these differences.

**Figure 4:**
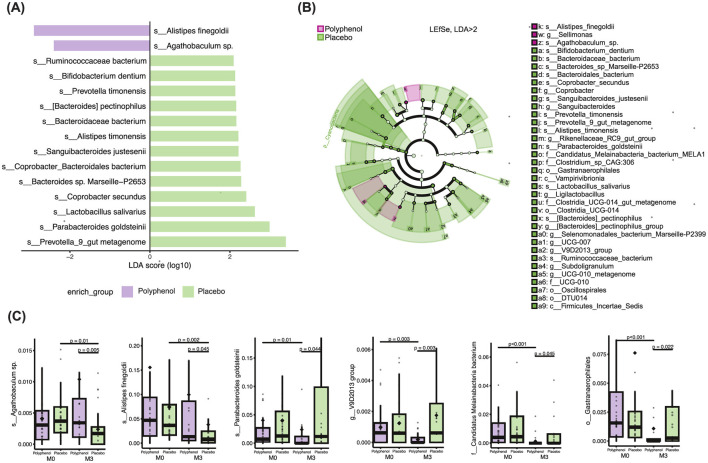
Gut microbiome composition changes based on LEfSe analysis following polyphenol supplementation in STEMI patients. **(A)** LEfSe analysis highlights differences in gut microbiota between the Placebo and Polyphenol groups 3 months after treatment. Biomarkers were identified using an LDA score > 2 at the species level, indicating significant taxa contributing to group differences. **(B)** The cladogram visually represents the differentially abundant bacterial species across various taxonomic levels. The root of the cladogram corresponds to the kingdom Bacteria, with concentric circles representing the hierarchical taxonomic levels from phylum to species. **(C)** Pairwise comparisons of six selected taxa between the groups and time points illustrate specific changes in microbial abundance due to the intervention. The *p*-value ≤ 0.05 indicates a significant difference.

Linear discriminant analysis effect size analysis (LefSe) indicating significant taxonomic changes (LDA > 2, *p* ≤ 0.05) identified several discrepant microbial taxa in the polyphenol and placebo groups at M3 point (Figures 4A, B).

Specific taxa dominated in the polyphenol group compared to the placebo group: species *Agathobaculum* sp., *p* = 0.004, effect size = 2.97; *Alistipes finegoldii, p* = 0.04, effect size = 2.44; and genus *Sellimonas, p* = 0.002., effect size = 2.35. On the contrary, species *Alistipes timonensis, p* = 0.003, effect size = 2.28; *Coprobacter secundus, p* = 0.006, effect size = 2.37; *Lactobacillus salivarius, p* = 0.01, effect size 2.6; *Parabacteroides goldsteinii, p* = 0.04, effect size = 2.9; *Prevotella timonensis, p* = 0.02, effect size =2.11; *Bifidobacterium dentium, p* = 0.02, effect size = 2.09; *Sanguibacteroides justesenii, p* = 0.03, effect size 2.2; *Bacteroides pectinophilus, p* = 0.04, effect size 2.1; *Ruminococcaceae bacterium, p* = 0.04, effect size = 2.1; genera *Prevotella, p* = 0.01, effect size 3.4, *Ligilactobacillus, p* = 0.04, effect size 2.7; *Sanguibacteroides, p* = 0.03, effect size = 2.2; *Coprobacter, p* = 0.01, effect size = 2.7 etc. were enriched in the placebo group, and depleted in polyphenol group, respectively (Figures 4A, B). The specific bacterial taxa identified by LefSe analysis showed significant differences not only between study groups but also within each group (polyphenol or placebo) from baseline to 3 months, highlighting distinct patterns of microbial changes (*p* ≤ 0.05) (Figure 4C). Overall, our LEfSe analysis demonstrated domination of various taxa from the *Firmicutes, Bacteroidetes, Cyanobacteriota* and *Actinobacteria phyla* in the placebo group, while only a few taxa in the Polyphenol group. The remaining Lefse results are presented in Supplementary Tables S3–S5.

### Correlation analysis between microbiome and clinical parameters

**Figure 5:**
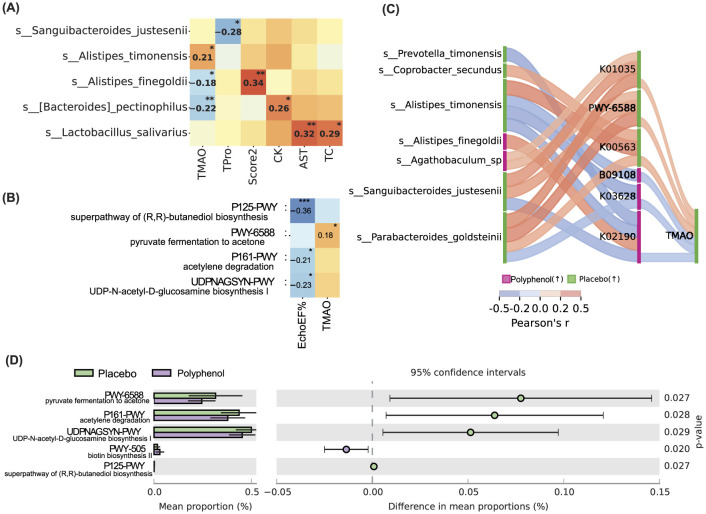
Association between clinical, taxonomic and functional markers at timepoint M3. **(A)** Correlation heatmap. Correlation between LEfSe markers and clinical parameters. **(B)** Correlation between STAMP markers and clinical parameters. **(C)** Sankey diagram. Correlation between significant features—LEfSe, STAMP markers and clinical parameters. KEGG and MetaCys functional data. **(A–C)** Pearson's r, FDR for all comparisons, no FDR when comparing between significant markers. Both baseline M0 and M3 data were used for correlation analysis. PWY-6588:pyruvate fermentation to acetone | MetaCys (*r* = 18, *p* = 0.0474); B09108 Metabolism of cofactors and vitamins | KEGG B (*r* = −19, *p* = 0.0314); K00563 rlmA1; 23S rRNA (guanine745-N1)-methyltransferase [EC:2.1.1.187] | KEGG D (*r* = 19, *p* = 0.03); K03628 rho; transcription termination factor Rho | KEGG D (*r* = −19, *p* = 0.034); K02190 cbiK; sirohydrochlorin cobaltochelatase [EC:4.99.1.3] | KEGG D (*r* = −18, *p* = 0.0477); K01035 atoA; acetate CoA/acetoacetate CoA-transferase beta subunit [EC:2.8.3.8 2.8.3.9] | KEGG D (*r* = 0.17, *p* = 0.0543); K01034 atoD; acetate CoA/acetoacetate CoA-transferase alfa subunit [EC:2.8.3.8 2.8.3.9] | KEGG D (*r* = 18, *p* = 0.0391); **(D)** STAMP functional markers at M3. MetaCys data. STAMP, *p* ≤ 0.05, non-overlapping 95CI. * < 0.05, ** < 0.01, *** < 0.001.

Correlation analysis between the taxa and clinical-laboratory parameters demonstrated significant relationships, thus negative correlations of s. *Alistipes finegoldy* (*p* = 0.04), s. *B. pectinophilus* (*p* = 0.01) with TMAO were observed, whilst s. *Alistipes timonensis* demonstrated a positive correlation with TMAO (*p* = 0.01). Regarding the rest of the positive correlations, s. *Lactobacillus salivarius* positively correlated with AST (*p* = 0.009) and TC (*p* = 0.03), s. *B. pectinophilus* with CK (*p* = 0.05), s. *A. finegoldy* with SCORE 2 (*p* = 0.003), respectively. s. *S. justesenii* negatively correlated with TPro (*p* = 0.02) (Figure 5A and Supplementary Table S6).

In addition, analysis of the predicted correlation between metabolic pathways and clinical-laboratory parameters demonstrated a few significant relationships. In contrast, P125-PWY-super pathway of (R, R)-butanediol biosynthesis, *p* = 0.0002, P161-PWY-acetylene degradation, *p* = 0.02, UDPNAGSYN-PWY-UDP-N-acetyl-D-glucosamine biosynthesis I, *p* = 0.02, demonstrated a significant negative correlation with EchoEF, PWY-6588-pyruvate fermentation to acetone demonstrated positive correlation with TMAO, *p* = 0.04 (Figure 5B and Supplementary Table S7).

### Metabolic pathway analysis

Further, the Sankey plot illustrating the networks between LEfSe markers, metabolic pathways, and TMAO levels showed that specific taxa nodes demonstrated various flows to nodes of metabolic pathways and connected to TMAO in two study groups. *s_Parabacteroides_goldsteinii* increased in the placebo group and decreased in the polyphenol group, respectively, appears to have strong positive correlations with several metabolic pathways (K01035, PWY-6588, K00563) and with TMAO in the placebo group, as well as negative correlations with K03628 and TMAO, particularly under the polyphenol condition. *s_Alistipes_timonensis* showed a mix of positive with K00563 and negative correlations with B09108, K03628, and K02190 metabolic pathways; a notable one is complex negative connections with B09108 and with TMAO under the polyphenol condition. s. *S. justesenii* also demonstrated complex relationships, a remarkable negative correlation with K02190 and TMAO level under the polyphenol condition (Figure 5C). *Alistipes finegoldii* and *Prevotella timonensis* demonstrated opposite correlations with K02190, positive and negative, respectively, and *Coprobacter secundus* positively correlated with K00563 (Figure 5C). Although the observed correlations were relatively weak and moderate (Pearson's r ranging from −0.18 to 0.36), they were statistically significant and can be associated with the biological complexity of microbial-metabolite interactions.

Thus, the analysis of metabolic pathways between two study groups at M3 was conducted. Three pathways, PWY-6588 pyruvate fermentation to acetone (*p* = 0.027), P161-PWY-acetylene degradation (anaerobic), (*p* = 0.028), UDPNAGSYN-PWY-UDP-N-acetyl-D-glucosamine biosynthesis (*p* = 0.029) were significantly lower in polyphenol group comparing to the placebo group. In contrast, PWY-5005 biotin biosynthesis biotin was higher in the polyphenol group compared to the placebo group (*p* = 0.02) (Figure 5D).

## Discussion

The gut-heart axis introduces perspectives in cardiology and suggests potential treatment approaches for CVD, including STEMI (37). Recent scientific directions focus on understanding the interaction between diet, gut microbiome, and cardiovascular disease (2, 3, 8, 27, 32, 38). Our study performed a randomized, placebo-controlled design to investigate how polyphenols may influence the gut microbiome and their potential association with STEMI.

Our findings suggest beneficial effects of polyphenol supplementation on gut microbiome composition in STEMI patients. The observation that TMAO levels remained stable in the polyphenol group while increasing in the placebo group appears consistent with previous research examining polyphenols in CVD management (19, 21, 22, 38) and MI (26). These observations correspond with several studies that reported possible polyphenol-mediated microbiome alterations that may affect TMAO levels (3, 39–42). A review by Leng et al. examining TMAO research highlights three main areas, such as TMAO's role in atherosclerosis mechanisms, its potential as a cardiovascular risk marker, and treatment approaches for TMAO reduction (3). Lombardo et al. found that diets rich in polyphenols may be associated with lower TMAO concentrations in blood and urine (42). Animal research indicates that plant-derived polyphenols might help reduce atherosclerosis by affecting gut microbiota composition and TMAO levels (30). We also observed improvement in liver function in the form of ALT decrease following polyphenol supplementation, which is notable considering the role of reduced FMO3 activity in the liver attenuating TMAO production from dietary precursors (43). While these data appear to be in line with our observations, larger studies are needed to confirm these preliminary results.

Further, we observed a decrease in the *Firmicutes*/*Bacteroidetes* ratio in the polyphenol group (*p* = 0.04), which may be of interest since TMAO-producing bacteria are often found within *Firmicutes, Proteobacteria* and *Actinobacteria phyla* (15, 16). Our findings align with studies proposing *F*/*B* as a potential indicator of gut dysbiosis (44) with some studies suggesting associations between higher *F*/*B* ratio and atherosclerosis (45), coronary artery disease (46), STEMI (47) and lower ratio with reduced cardiovascular risk factors (24, 48). According to a study by Cho et al., individuals with a higher *F*/*B* ratio showed greater TMAO production from precursors (49). While our findings appear consistent with a previous animal study showing a decreased *F*/*B* ratio after polyphenol administration (50) and human studies of the gut microbiome in metabolic syndrome after red wine consumption (51), the clinical relevance of these changes requires validation in more extensive studies.

Next, our study showed a decrease in microbiome diversity within both polyphenol and placebo groups between baseline and three-month time points, which appears to coordinate with studies on metabolic diseases and CVD (24) but differs from findings by Istas et al. focused on polyphenols and vascular function, which did not identify significant changes after polyphenol intake (23).

Regarding microbial abundance, we observed a decrease in Bacteroides in both groups over the study period, which differs from some previous studies (52, 53). Most et al. found that polyphenol supplementation decreased the abundance of *Bacteroidetes* in men (54), which parallels our observation in both groups, though our results suggest this may be independent of polyphenol intervention. We also observed an increase in *Roseburia* abundance in the polyphenol group after 3 months of polyphenol intake, a bacterium known for its butyrate-producing ability and anti-inflammatory, probiotic properties (55–57).

The LEfSe analysis in our study demonstrated enrichment of *Agathobaculum* sp.*, Alistipes finegoldii* and *Sellimonas* in the polyphenol group, compared to the placebo group at M3. *Agathobaculum* produces butyrate and has been associated with gut homeostasis and barrier function (58, 59) and potentially with cognitive function (59, 60). These findings correspond to research on STEMI patients, and animal models revealed enrichment of butyrate-producing bacteria within the first 3 days after the injury as a compensatory adaptive response. However, this trend was not preserved after 28 days. Regarding specific butyrate-producing taxa, this study reports that *Butyricimonas virosa, Anaerotruncus, Alistipes, Holdemanella, Subdoligranulum, Butyricicoccus* were enriched after injury in human STEMI patient samples, and *Faecalibacterium, Roseburia* enriched in the non-human primate model (47), which partially corresponds with our findings. Moreover, butyrate administration and colonization with butyrate-producing bacteria improved cardiac function, reduced inflammation, and inhibited sympathetic neural remodeling after MI in animal studies (47, 61).

Intriguing results were obtained from representatives of the genus *Alistipes*; we found that *Alistipes finegoldii* increased in the polyphenol group, while *Alistipes timonensis* increased in the placebo group. This observation aligns with previous research suggesting different beneficial effects of these bacteria in CVD (62), while other research has associated *Alistipes* with various disorders (63). Additionally, some species of *Alistipes* are known as butyrate producers, with increased levels upon probiotics intake, proposing its anti-inflammatory benefits (64). Our results of correlation analysis demonstrate opposite relationships; precisely, a negative correlation of *A. finegoldy* and a positive correlation of *Alistipes* timonensis with TMAO corresponds to the information above. *Sellimonas* demonstrated a protective role in endocarditis (65) and is proposed as a possible indicator of gut homeostasis. It is also known for its involvement in butyric acid production (66). In line with our observations, previous animal studies have shown that polyphenol supplementation is linked to alterations in microbial communities, specifically showing elevated levels of butyrate-producing bacteria from the *Lachnospiraceae* family (*Agathobaculum* and *Roseburia*) (67). Although we did not observe significant increases in *Bacteroides, Lactobacillus, Bifidobacterium* from *Verrucomicrobia* phylum, and bacteria from *Akkermansia* genus as was previously reported (68), our results align with the general effect of polyphenol increasing abundance of SCFA-producing bacteria (32). The enrichment of short-chain fatty acid (SCFA)-producing bacteria observed in our polyphenol group likely plays a significant role in maintaining stable TMAO levels. Chen et al. described how various butyrate-producing bacteria contribute to cardiovascular health by mediating the gut-heart axis (69). According to existing data butyrate functions as both a primary energy source for intestinal epithelial cells and a critical regulator of gut barrier integrity, limiting the translocation of harmful metabolites like TMA into bloodstream (69). Our observation of increased *Roseburia, Agathobaculum, Alistipes finegoldii*, and *Sellimonas* populations correlates with the beneficial bacteria Chen et al. identified as protective against atherosclerosis. These bacteria can competitively exclude TMAO-producing bacteria in the gut ecosystem (70), influence lipid metabolism through multiple mechanisms, including enhanced ABCA1 expression via GPR109A activation and HDAC inhibition, leading to improved cholesterol efflux from macrophages (69), improved hepatic metabolic condition via the Free Fatty Acid Receptors 3 (FFAR3) affecting lipids synthesis (71), as evidenced in our study by reduced ALT and AST levels in polyphenol group, and stable LDL levels in polyphenol group compared to increased LDL in placebo group. These cardioprotective effects are further supported by Avendano-Ortiz et al.'s findings that butyrate levels are significantly decreased during acute myocardial infarction but normalize during recovery (72). Recent study by Cheng et al. demonstrates that butyrate directly ameliorates TMAO-induced cardiac effects (73). Collectively, these studies suggest that polyphenol supplementation maintains a favorable gut microbiome profile that supports cardiovascular health through enhanced butyrate production.

Our LEfSe analysis also revealed a distinctive microbial signature in the Placebo group, characterized by the enrichment of various bacterial taxa predominantly from *Firmicutes, Bacteroidetes*, and *Cyanobacteria phyla*, respectively demonstrating the depletion of these bacteria in the polyphenol group. Interestingly, according to available data, certain representatives of these phyla utilize enzymes (CutC/D and YeaW/X) to metabolize dietary choline and carnitine, converting them to TMA as part of their energy-generating metabolic pathways (74, 75). The most significantly enriched species included *P. goldsteinii* and various members of *Bacteroidales* and *Oscillospirales* orders. Interestingly, this microbial pattern differs from previously reported signatures in STEMI patients. Kwun et al. described increased abundance of *Proteobacteria* and *Enterobacteriaceae* in STEMI patients (76), while Prins et al. identified positive associations between cardiovascular risk and species such as *Collinsella stercoris* and *Flavonifractor plautii* (77). These species were not significantly enriched in our Placebo group. Notably, *P. goldsteinii* has shown associations with high-fat diet feeding and obesity-related parameters in some studies (78, 79), showing a probable link of this bacteria to lipids metabolism, possibly contributing to growth TMAO production's precursors. Among other dominant taxa in the placebo group, *Coprobacter secundus* has been previously associated with various diseases (80). Our finding of lower *Lactobacillus* abundance in the polyphenol group differs from some previous studies (52, 81). While this genus is generally considered beneficial, it has been associated with certain conditions when underlying factors are present (82); for instance, an increased abundance of some *Lactobacillus* species has been reported in rheumatoid arthritis (83). Our results show a positive correlation between *Lactobacillus salivarius* and TC level. Overall, the polyphenol group demonstrates a more selective microbiome profile with a few dominated beneficial bacteria, in contrast to the placebo group's more diverse dominant bacterial distribution with multiple phyla, including *Firmicutes* and *Actinobacteria* representatives, which corresponds with previous studies related to TMAO-producing bacteria and polyphenol effects (2). The distinct microbiome profiles between polyphenol and placebo groups in our study suggest that polyphenols may selectively modulate bacterial composition, potentially creating a more focused microbiome environment. Nevertheless, the functional consequences of these changes require additional metabolic, functional, or metagenomic analyses.

Further, our predicted pathway analysis suggests potential mechanisms that might contribute to the observed differences in TMAO levels between groups. We hypothesize that the polyphenol group's reduced representation of several metabolic pathways compared to placebo—such as PWY-6588 (pyruvate fermentation to acetone), P161-PWY (acetylene degradation), and UDPNAGSYN-PWY (UDP-N-acetyl-D-glucosamine biosynthesis)—may reflect alterations in bacterial metabolism that could influence TMA production. PWY-6588 (pyruvate fermentation to acetone), which produces ketone bodies that, while essential for normal cardiac energy metabolism (84, 85), have been associated with certain cardiovascular conditions in several studies (84, 86–88). De Koning et al. observed that elevated ketone body levels in STEMI patients at 24 h were linked to worse outcomes, including more considerable heart damage and lower left ventricular function, and these levels remained high for up to 4 months (89). Moreover, acetone produced via this pathway could serve as a carbon source for bacteria and influence gut pH (90).

According to some research, P161-PWY (acetylene degradation) produces acetate that may affect myocardial function, inhibiting myocardial contraction and precisely affecting the SCFA receptor GPR43 (91, 92). UDPNAGSYN-PWY (UDP-N-acetyl-D-glucosamine biosynthesis), a component in bacterial cell walls, including *Escherichia coli* (93), may be indirectly linked to a previous study by Yoo et al., suggesting that high-fat diets can modify the gut environment, facilitating *E. coli* growth and its respiratory-dependent choline catabolism, leading to TMAO increase (94). However, our study did not observe significant changes in *E. coli* abundance, indicating that the relationship between dietary interventions, bacterial metabolism, and TMAO production may be more complex than previously hypothesized. The observed decreases in energy metabolism pathways negatively correlating with ejection fraction can be hypothesized as a beneficial adaptive response after STEMI, as reduced myocardial energy metabolism decreases oxygen demand, limits injury, prevents adverse remodeling, and reduces mechanical stress on the healing myocardium. Conversely, the elevated PWY-5005 (biotin biosynthesis) in the polyphenol group might be relevant to epithelial health and barrier function (95, 96), host lipid metabolism, and gene expression (97), potentially affecting the absorption of TMA or its precursors. However, we acknowledge that these proposed mechanisms are preliminary and require further investigation to establish causal relationships.

Our Sankey plot analysis supports the complex interplay between these mechanisms and TMAO levels. For instance, we observed a decrease in *Parabacteroides goldsteinii* abundance in the polyphenol group and its positive correlation with the PWY-6588 pathway and TMAO levels. Yet, our analysis suggests potential relationships between specific bacteria, metabolic pathways, and TMAO levels, though these mechanisms need further clarification, warranting further research.

Based on our preliminary findings and existing literature, polyphenols demonstrate a targeted approach to TMAO metabolism by restructuring gut microbiota and reducing the *Firmicutes*/*Bacteroidetes* ratio, probably targeting bacteria involved in TMAO production. This microbial shift resulted in decreased abundance of bacterial taxa utilizing specific enzymes converting dietary precursors to TMA. Moreover, butyrate-producing bacteria increased in the polyphenol group, can competitively inhibit TMAO-producers, strengthen intestinal barrier function and reduce the transfer of TMA into the circulation, additionally, demonstrating protective effects against TMAO-induced cardiac damage, impacting liver and lipids metabolism.

While our observations suggest that polyphenols may help maintain stable TMAO levels in patients with STEMI through potential microbiome modulation, these findings should be considered preliminary. Further extended research with larger sample sizes is needed to confirm these results and determine their clinical significance.

## Conclusion

Our study suggests a beneficial association between polyphenol supplementation, gut microbiome composition, and stable TMAO levels in STEMI patients. We observed significant changes in bacterial diversity, *F*/*B* ratio, and specific taxa, particularly butyrate-producing bacteria. These microbiome alterations may contribute to the stability of the TMAO level seen in the polyphenol compared to the increase observed in the placebo group. Obtained results support potential mechanisms linking polyphenol intake to microbiome modulation and cardioprotective effect. While promising, these preliminary findings require further validation to establish their significance for cardiovascular health through clinical trials with larger sample sizes, longer study durations, and more advanced sequencing methods, such as shotgun, metatranscriptomics, and metabolomics.

## Limitations

This study has several limitations. First, the sample size was relatively small, which may affect the generalizability of the findings. Future studies with larger cohorts must validate the findings and ensure they can be applied broadly across different demographics. Second, we used 16S rRNA sequencing, limiting microbial identification and functional analysis depth. A more comprehensive approach, such as shotgun metagenomics, metatranscriptomics, and metabolomics, would provide a deeper understanding of the microbial community and the specific gene functions involved, enabling more accurate data into the mechanisms by which polyphenols influence the gut microbiome and cardiovascular health. Third, the duration of the polyphenol supplementation was limited to 3 months. This may not have been long enough to capture the full extent of the long-term effects of polyphenol intake on the gut microbiome or its sustained impact on the cardiovascular system. Longer-term studies are required to assess whether the observed changes in microbiome composition and TMAO levels are maintained over time and whether they translate into continuing cardiovascular benefits. Lastly, we acknowledge that one of the study limitations was the lack of dietary control, as the research extended beyond the hospitalization period. While baseline homogeneity in laboratory parameters and microbiome diversity demonstrates the absence of significant dietary differences between groups that could have confounded our results, our study did not fully account for potential lifestyle and dietary variations among participants that could independently affect gut microbiome composition beyond the polyphenol intervention. Future research should control for these variables to establish more definitive conclusions about polyphenol supplementation effects.

## Data Availability

The datasets presented in this study can be found in online repositories. The names of the repository/repositories and accession number(s) can be found below: https://zenodo.org/records/13836920, Zenodo.
